# Fusarium Wilt of Banana: Current Knowledge on Epidemiology and Research Needs Toward Sustainable Disease Management

**DOI:** 10.3389/fpls.2018.01468

**Published:** 2018-10-19

**Authors:** Miguel Dita, Marcia Barquero, Daniel Heck, Eduardo S. G. Mizubuti, Charles P. Staver

**Affiliations:** ^1^Embrapa Mandioca e Fruticultura, Bahia, Brazil; ^2^Bioversity International, Montpellier, France; ^3^Institute of Environment, Natural Resources and Biodiversity, Universidad de León, León, Spain; ^4^Departamento de Fitopatologia, Universidade Federal de Viçosa, Viçosa, Brazil

**Keywords:** *Musa* spp, panama disease, *Fusarium oxysporum* f. sp. *cubense*, integrated pest management, epidemiology

## Abstract

Banana production is seriously threatened by Fusarium wilt (FW), a disease caused by the soil-borne fungus *Fusarium oxysporum* f. sp. *cubense* (*Foc*). In the mid-twentieth century FW, also known as “Panama disease”, wiped out the Gros Michel banana industry in Central America. The devastation caused by *Foc* race 1 was mitigated by a shift to resistant Cavendish cultivars, which are currently the source of 99% of banana exports. However, a new strain of *Foc*, the tropical race 4 (TR4), attacks Cavendish clones and a diverse range of other banana varieties. *Foc* TR4 has been restricted to East and parts of Southeast Asia for more than 20 years, but since 2010 the disease has spread westward into five additional countries in Southeast and South Asia (Vietnam, Laos, Myanmar, India, and Pakistan) and at the transcontinental level into the Middle East (Oman, Jordan, Lebanon, and Israel) and Africa (Mozambique). The spread of *Foc* TR4 is of great concern due to the limited knowledge about key aspects of disease epidemiology and the lack of effective management models, including resistant varieties and soil management approaches. In this review we summarize the current knowledge on the epidemiology of FW of banana, highlighting knowledge gaps in pathogen survival and dispersal, factors driving disease intensity, soil and plant microbiome and the dynamics of the disease. Comparisons with FW in other crops were also made to indicate possible differences and commonalities. Our current understanding of the role of main biotic and abiotic factors on disease intensity is reviewed, highlighting research needs and futures directions. Finally, a set of practices and their impact on disease intensity are discussed and proposed as an integrative management approach that could eventually be used by a range of users, including plant protection organizations, researchers, extension workers and growers.

## Introduction

Bananas, the world's most important fruit in terms of production volume and trade (FAOSTAT, 2017) and among the world's top 10 staple foods, is seriously threatened by Fusarium wilt (FW). The disease, considered one of the most destructive banana diseases in history (Stover and Simmonds, 1987), is caused by *Fusarium oxysporum* f. sp. *cubense* (E.F. Smith) W.C. Snyder & H.N. Hansen (hereafter referred as *Foc)*. A soil-borne pathogen with an extremely long residence time in soil, *Foc* infects the xylem, induces wilt and kills banana plants (Stover R., 1962). The pathogen was first reported in Australia (Bancroft, 1876; Ploetz and Pegg, 1997) and has been spreading globally with the informal exchange of planting material and the movement of spore-bearing soil (Ploetz, 2015a,b). The expansion of export banana in large monocropped fields in the Americas from the early 1900's was based on practices favorable to disease spread. By the mid-twentieth century the banana industry in the Americas, based in the susceptible cultivar Gros Michel (AAA) was in crisis due to FW (Stover R., 1962; Ploetz, 2005). A highly successful shift to FW-resistant cultivars of the Cavendish (AAA) subgroup, contributed to the expansion of Cavendish production for both national and export markets, but also resulted in a dramatic decline in FW research. The appearance of a new race of *Foc*, to which Cavendish and many other cultivars are highly susceptible, has generated a global concern and new demands for solution-oriented research on FW of banana. New resistant cultivars are not foreseen in the short-term, although clonally selected varieties with partial resistance have shown some promise (Hwang and Ko, 2004). Thus, research on epidemiology-based management programs has again become a high priority, which this review proposes to address.

*Foc*, a highly variable pathogen, is comprised of different evolutionary lineages (O'Donnell et al., 2009). At least 24 vegetative compatibility groups (VCGs) are known to date in *Foc* (Ploetz and Correll, 1988; Fourie et al., 2011; Mostert et al., 2017), which can affect *Musa acuminata, M. balbisiana, M. schizocarpa*, and *M. textilis* (Musaceae: Zingiberales; Ploetz, 2015b). Different races of the pathogen are identified based on the pathogenicity to reference host cultivars: race 1 (R1) affects Gros Michel (AAA) and Manzano/Apple/Latundan (Silk, AAB); race 2 (R2) affects cooking bananas of the Bluggoe (ABB) subgroup and race 4 (R4) affects all cultivars in the Cavendish (AAA) subgroup in addition to those susceptible to R1 and R2 (Waite and Stover, 1960; Su et al., 1986). A pathogen population causing FW in *Heliconia* spp., was described as race 3, but it is no longer considered as part of *Foc* (Ploetz, 2005).

Fusarium wilt epidemics occurring in Gros Michel, the original export banana, were attributed to *Foc* R1 (Stover R., 1962). The substitution of Gros Michel by Cavendish cultivars, resistant to *Foc* R1, though at high economic costs, solved the problem (Ploetz, 2006) and since then the banana export industry has expanded rapidly based on Cavendish. However, when cultivated under seasonal abiotic stresses (mainly low temperature) in subtropical regions such as South Africa, the Canary Islands and parts of Australia, Cavendish cultivars were susceptible to *Foc* R4 (Su et al., 1986; Ploetz and Pegg, 1997).

Until 1989, *Foc* was only reported affecting Cavendish in subtropical regions. However, a new variant that severely affects Cavendish cultivars in the tropics was reported in 1990 (Ploetz and Pegg, 2000; Ploetz, 2006). To discriminate *Foc* populations that only affect Cavendish in the subtropics from the populations that affect Cavendish in the tropics, two divisions of *Foc* R4 were created: Subtropical race 4 (SR4) and tropical race 4 (TR4; Ploetz, 2006). While *Foc* SR4 causes disease in Cavendish only in the subtropics, *Foc* TR4 is pathogenic under both tropical and subtropical conditions (Buddenhagen, 2009; Mostert et al., 2017). In addition, different VCGs (0120, 01201, 01202, 01209, 01210, 01211, 01215, 0120/15; 0129/11) have been associated with *Foc* SR4, while only one VCG (01213/16) to *Foc* TR4 (Buddenhagen, 2009; Mostert et al., 2017).

**Figure 1:**
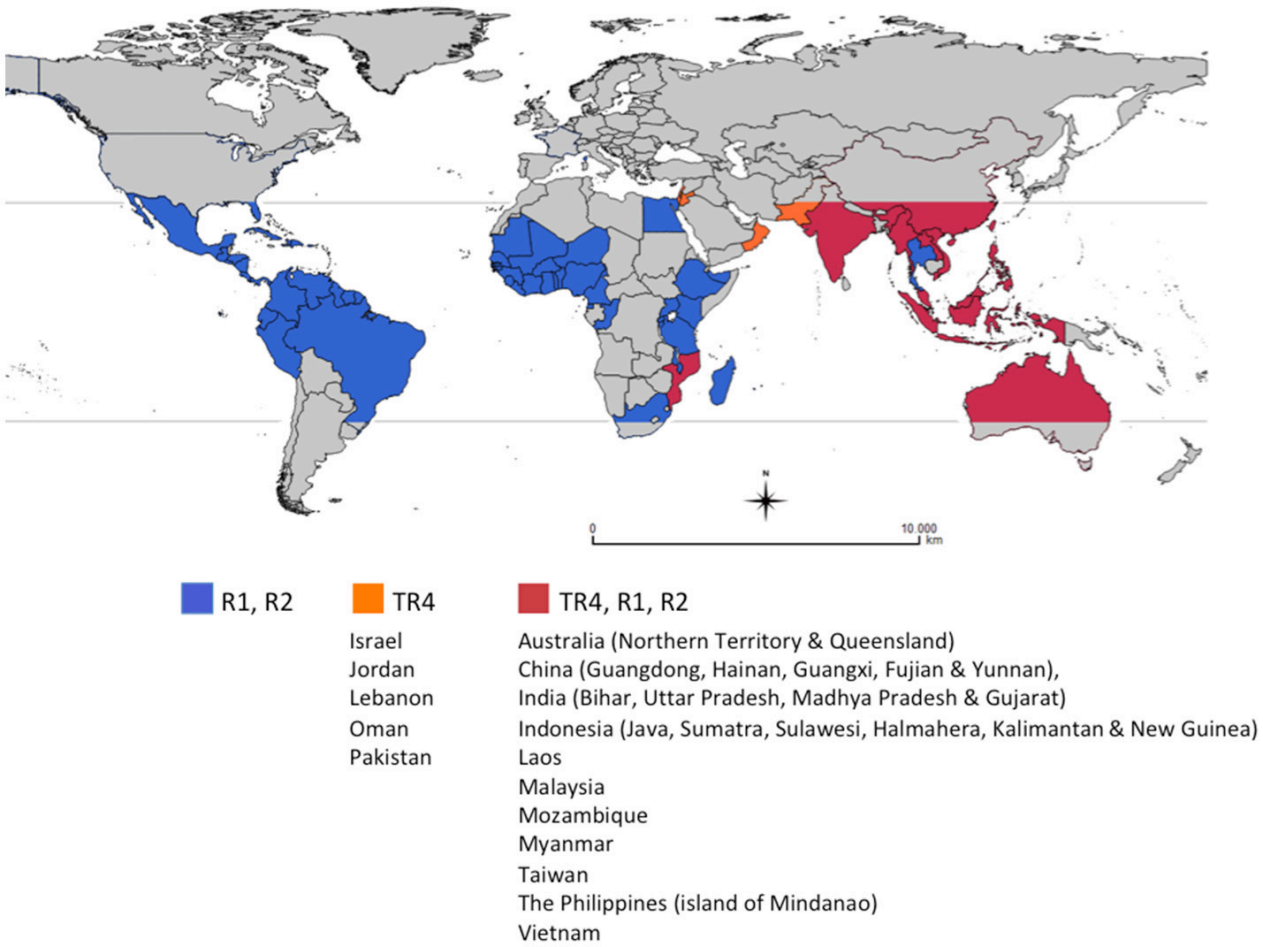
Global distribution of races of *Fusarium oxysporum* f. sp. *cubense (Foc)*, causal agent of Fusarium wilt of banana. This map considers producing countries with presence or absence of a given race of *Foc* and does not represent banana-producing areas by countries. R1: Race 1, R2: Race 2, TR4: Tropical race 4. Races 1 (R1) and 2 (R2) are widely distributed in banana producing countries affecting local varieties (see introduction for more details). Subtropical race 4 was not included as it corresponds to *Foc* populations present in subtropical producing areas in Australia, Brazil, Canary Islands, China, South Africa and Taiwan, causing intermittent yield losses in Cavendish cultivars. For more information on the distribution of TR4 see http://www.promusa.org/Tropical+race+4+-+TR4#Distribution.

For more than 20 years, *Foc TR4* was restricted to East and parts of Southeast Asia and the Northern Territory of Australia, but recent reports confirmed its presence in Jordan, Oman, Mozambique (2013), Lebanon, Pakistan (2015) (García-Bastidas et al., 2014; Ordoñez et al., 2015), Vietnam (Hung et al., 2018), Laos (Chittarath et al., 2018), Myanmar (Zheng et al., 2018), and Israel (Maymon et al., 2018). In Australia, *Foc* TR4 was reported in the Northern Territory since 1997 (Bentley et al., 2001; Conde and Itkethley, 2001), but new outbreaks were reported in Queensland in 2015 (O'Neill et al., 2016). There are also informal reports that *Foc* TR4 is also present in India (Figure 1).

In 28 years *Foc* TR4 has been moved by planting material, soil vectored by workers, vehicles and planting materials, irrigation, and floodwaters resulting in the loss of hundreds of thousands of hectares of Cavendish production across China and into northern Southeast Asia and in the Philippines and Indonesia. This on-going dissemination of *Foc* TR4 and the impact caused in Cavendish in the region highlight the major threat that this race represents both to countries where the disease is already present and still spreading and those still TR4-free (Figure 1).

Although *Foc* TR4 has been highly associated with Cavendish monoculture, this pathogen also affects many other cultivars important for food security and income generation (Hermanto et al., 2011; Mostert et al., 2017). Producers facing TR4 and even producers of key cultivars for national markets susceptible to R1, face the spread and build-up of FW and resulting threat to income. In practice, cultivars like Gros Michel, Pome, Bluggoe, Pisang Awak and many South Pacific plantains, continue to be grown and marketed through two mechanisms. The production and supply of susceptible banana varieties is mainly due to movement of banana growers and establishment of new fields in clean areas.

The frequent claim that effective management of FW can only be achieved with resistant cultivars is partly due to the success of Cavendish for *Foc* R1. Chemical, biological and cultural practices have always been downplayed as not effective (Ploetz, 2015b). However, quantitative resistance linked to cultural and biological practices have been effective in different scenarios allowing farmers to grow susceptible varieties. In Brazil, for instance, Prata-type cultivars have high market value and are susceptible to FW, but combining resistance with cultural practices growers can cope with the disease and make the banana crop profitable (Haddad et al., 2018). Growers in Colombia have also adopted a two-cycle strategy to produce Gros Michel, rotating production before pathogen inoculum builds up in the soil.

To date, most elements of the strategy to manage *Foc* TR4 are based on limited research conducted to understand epidemics caused by R1. The replacement of Gros Michel by a fully resistant cultivar, partially removed FW from the “list of banana constraints” and consequently research efforts on this disease in most countries practically stopped.

With the threat of *Foc* TR4, research efforts have revived. Many reviews cover general aspects of history, pathogen biology, epidemiology and management options (Ploetz and Pegg, 1997, 2000; Ploetz, 2005, 2006, 2015a,b; Daniells, 2011; Ghag et al., 2015). These sources are quite useful to understand the historical and basic epidemiological aspects of FW and how the banana industry has been impacted. In this review we summarize the current knowledge of FW epidemiology, such as pathogen survival, dispersal, and host invasion. Most importantly, we identify knowledge gaps and research questions on how to live with the spread of *Foc* TR4 and other races of *Foc*. The impact of biotic and abiotic factors and crop management practices on FW intensity are discussed. Finally, a set of practices and their impact on disease intensity are discussed and proposed as an integrative management approach.

## Pathogen survival

Most species of the *F. oxysporum* complex, including *Foc*, are able to survive in the absence of it primary host, mainly in the form of the thick-walled survival spores, chlamydospores (Stover R., 1962; Leslie and Summerell, 2006). Chlamydospores are resistant to desiccation, resilient in unfavorable environmental conditions (Stover R., 1962), and may survive in the soil for more than 20 years (Stover R., 1962; Buddenhagen, 2009). Contrary to what is commonly assumed, chlamydospores of *Foc* are constantly produced once the host is invaded even before external symptoms are visible (Li et al., 2011), and not just after the death of the banana plant.

The capacity of *Foc* to colonize and grow saprophytically in debris increases chlamydospore production and contributes to increased pathogen persistence in the soil (Stover and Waite, 1960; Stover R., 1962). In addition to chlamydospores, long-term survival of *Foc* is promoted through the infection of weeds and non-economic host plants (hereafter collectively referred to as weeds). Studies carried out in America and Australia revealed that some weed species can be colonized by *Foc* without visible symptoms. *Foc* R1 was found in *Paspalum fasciculatum, Panicum purpurescens, Ixophorus unisetus* (Poaceae), and *Commelina diffusa* (Commelinaceae) in Central America (Waite and Dunlap, 1953), while *Foc* SR4 was reported in *Paspalum* spp. and *Amaranthus* spp. (Amaranthaceae) in Australia (Pittaway et al., 1999). *Foc* TR4 was found in roots of *Chloris inflata* (Poaceae), *Euphorbia heterophylla* (Euphorbiaceae), *Cyanthillium cinereum* and *Tridax procumbens* (Asteraceae), growing in infested banana plantations in Australia (Hennessy et al., 2005). In all cases, weeds did not show external symptoms resembling FW. These findings suggest that *Foc* may be able to survive as endophyte in other hosts and when bananas are replanted in the area, weed hosts can act as FW inoculum reservoir.

The impact of weed management practices on *Foc* dispersal and survival needs further attention. Cover crops are commonly recommended in banana plantations as a soil-health practice to control weeds and nematodes, prevent soil erosion, and to reduce within field spread of FW (Charles, 1995; Fongod et al., 2010; Duyck et al., 2011; Djigal et al., 2012; Pattison et al., 2014; Tardy et al., 2015). However, no studies were found which investigate the potential role of cover crops used in banana as secondary hosts of *Foc*.

Little is known about the potential of *Foc* detected in weeds to infect banana, the infection process and related soil and abiotic factors that affect the efficacy of this potential source of inoculum, albeit the large impact these issues may potentially have for the epidemiology and management of FW. The life cycle of *Foc* in weeds or in alternative hosts appears not to be the same as in banana because no disease symptoms are observed, but what kind of *Foc* structures are present inside an alternative host? Are chlamydospores produced in these weeds or are they only produced when these plants die? Does chemical (herbicides) or mechanical (mowing) weed management stimulate the production of chlamydospores, as empirically claimed when banana plants are eliminated with glyphosate? Can chlamydospores originating from weedy vegetation survive in the soil and infect banana plants again? While sound scientific-based information is not available, the recommendation for using cover crops and weed management strategies in *Foc*-affected areas should consider its potential role as *Foc* propagators. Unfortunately, a drawback to answer these research questions is the lack of effective diagnostic tools with high resolution to take into account the worldwide diversity of *Foc*. Once these tools are available, relevant knowledge on these epidemiological aspects in different environments and *Foc* populations can be generated.

## Infection process, plant-pathogen interaction and disease development

**Figure 2:**
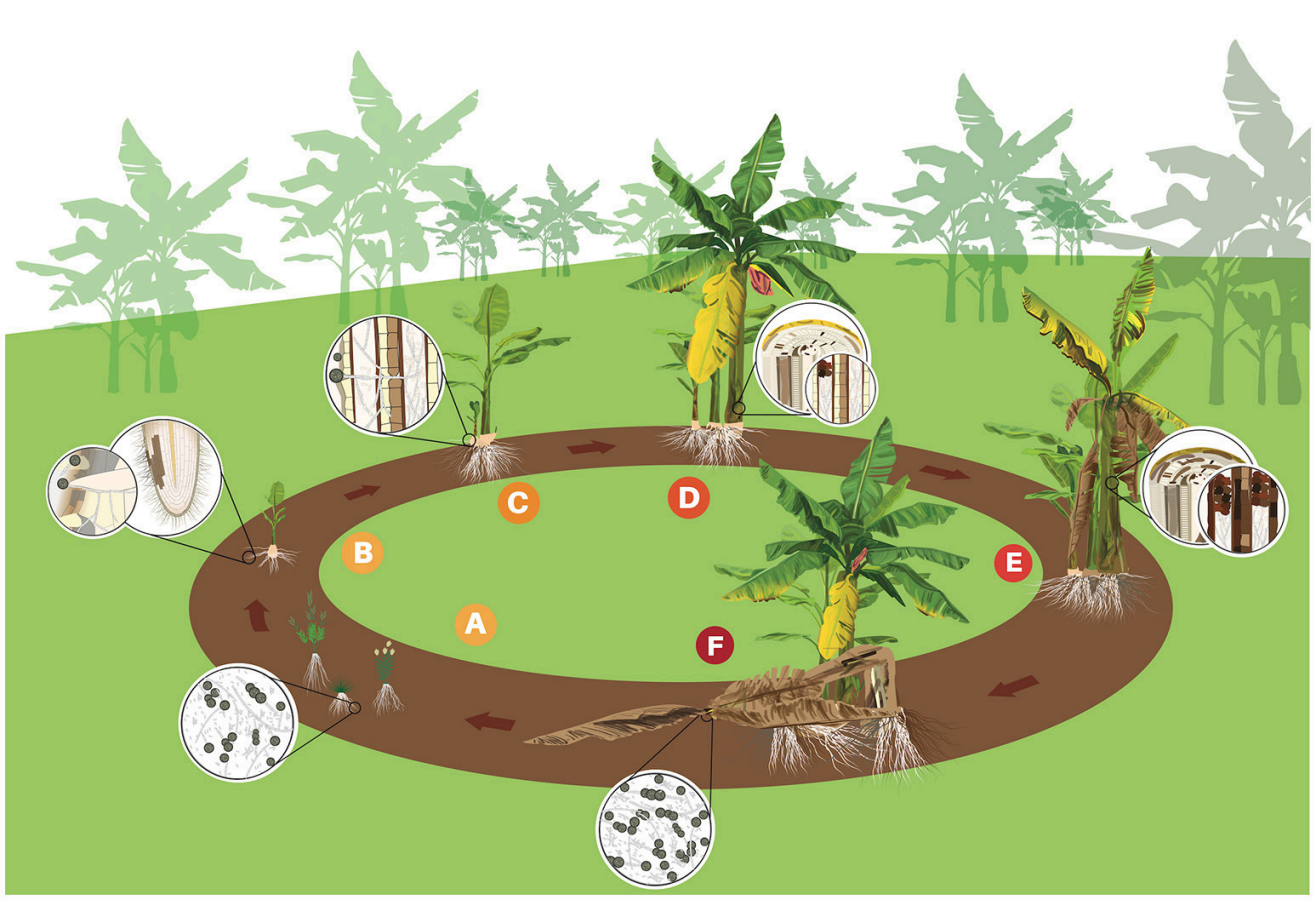
Life cycle of *Fusarium oxysporum* f. sp. *cubense (Foc)* in banana. **(A)** Spores (micro and macro conidia and chlamydospores) rest in the soil or on alternative hosts such as weeds. **(B)** Chlamydospores germinate stimulated by root exudates and the germ-tubes penetrate banana roots. **(C)** *Foc* grows through the cortex to the epidermis and mycelium invades the vascular system. **(D)** Conidia and chlamydospores are constantly produced in the vascular tissues. Conidia are rapidly distributed through the plant via transpiration system. Mycelium and gum blocks the vascular tissues and first symptoms of yellowing are observed in the older leaves. **(E)** *Foc* colonizes and destroys more vascular tissues provoking intense wilting. **(F)** Infected plant dies and the follower plant (daughter), which was contaminated by the mother plant through vascular connection, shows initial symptoms. Mother plant eventually falls down and disease cycle starts again.

In the soil, the different *formae specialis* of *F. oxysporum* show negligible capacity for self-movement or growth without a host tissue (Figure 2A; Rekah et al., 1999). *Foc* seems to be no exception, even though no solid experimental data to support this claim were found. The structures of *Foc* remain dormant until stimulated to germinate by host or non-host root exudates or by the direct contact with susceptible root tissues (Figure 2B; Stover R., 1962). Conidia and hyphae of *Foc* can be seen adhered to root surfaces at 1 or 2 days post inoculation (Guo et al., 2015; Li et al., 2017). Hyphae can grow along the grooves at junctions between epidermal root cells and colonize the root surface (Guo et al., 2015). Infection takes place through secondary or tertiary feeder roots, but not through the main root, unless the central core is exposed directly to pathogen structures (Trujillo and Snyder, 1963). Penetration can occurs either directly or through wounds (Figure 2B) and no true appressoria or penetration pegs have been observed (Li et al., 2011, 2017). Hyphae become swollen at penetration site, but return to their normal size afterwards (Li et al., 2011). Occasionally lesions develop at the site of initial infection, but more often they are located near the root base (Rishbeth, 1955).

Even when the pathogen successfully attaches its structures to the roots, most of the infection attempts seem to be blocked by the host (Underwood, 2012). The cell wall can be a source of nutrients for pathogens as well as a barrier to access a most suitable environment to their survival (Cantu et al., 2008). Plant pathogens must pass this barrier to complete the infection and interact with the host (Underwood, 2012). The cell wall is composed of complex polysaccharides (cellulose, hemicellulose, or pectin) with associated proteins and aromatic compounds (Caffall and Mohnen, 2009). Some of the major groups of cell wall pectins could be digested by a series of cell wall-degrading enzymes secreted by hemibiotrophic pathogens, such as pectin methylesterases (PMEs), polygalacturonases and polymethylgalacturonases (Cantu et al., 2008). In response to *Foc* infection, the cell wall of banana roots trigger a series of coordinated responses, such as pectin methylesterification (Ma et al., 2013) and changes in the spatial distribution and abundance of hydroxiproline-rich glycoproteins, PMEs and pectins (Fan et al., 2017; Wu et al., 2017), which have direct impact on the host resistance. Other defenses in banana against *Foc* are incited by salycilic acid and jasmonic acid/ethylene. Both pathways may involve systemic acquired resistance (Wu et al., 2013), DNA methylation (Luo et al., 2016), and changes in the expression of genes related to pathogenesis, transcription factors, hypersensitive reaction, and synthesis of phytoalexins (Bai et al., 2013; Li et al., 2013; Niu et al., 2018). Genes related to the cell wall biosynthesis (lignification or degradation) can also be involved in the resistance of banana against *Foc* (Bai et al., 2013; Niu et al., 2018).

*Fusarium oxysporum* f. sp. *cubense* has been considered a necrotrophic or hemibiotrophic pathogen. Apparently, *Foc* needs to interact during part of its life cycle with living plant cells. In addition to the strategies described in the previous paragraph, *Foc* evolved to have a diverse array of proteins that determine infection capacity in bananas, similar to *Fol* in tomato (Takken and Rep, 2010).

Differences in the composition of *SIX* (secreted in xylem) homologues genes, involved in pathogenicity of *F. oxysporum* (Rep et al., 2004; Meldrum et al., 2012), were also observed in *Foc* races. While a *Foc* R4 strain presented homologues of *SIX1, SIX7* and *SIX8*, only *SIX1* was present in a *Foc* R1 strain (Meldrum et al., 2012). An additional study showed that *Foc* TR4 had three copies of *SIX1* gene (*Six1a*-*Six1c*), one copy of *SIX2, SIX6* and *SIX8*, while only one copy of *SIX1* and *SIX6* were observed in *Foc* R1 (Guo et al., 2014). Recently, using a whole-genome sequencing approach, Czislowski et al. (2017) studied 23 VCGs of *Foc* and identified seven *SIX* genes (*SIX1, SIX2, SIX6, SIX8, SIX9, SIX10*, and *SIX13*). Strains belonging to R1 and R2 generally share the same profile of *SIX*. In contrast, strains of SR4 and TR4 presented more diverse profiles (Czislowski et al., 2017). The homologues, *SIX1* and *SIX9* were conserved in all VCGs, while no *SIX* genes were observed in a non-pathogenic *F. oxysporum* strain analyzed (Czislowski et al., 2017).

Transcriptome analysis revealed more virulence-associated genes up-regulated in *Foc* R4 than in R1, suggesting a more active pathogenesis in R4 interactions (Guo et al., 2014, 2015). While the role of *SIX* genes in pathogenicity against banana needs to be further demonstrated, the differences between *Foc* TR4 and R1 regarding composition and copy numbers of *SIX* genes seems to be a plausible hypothesis for their differential virulence patterns. However, comparisons between *Foc* R1 isolates regarding *SIX* genes need to consider that they can belong to different VCGs and lineages in spite of being classified as the same race. Czislowski et al. (2017) observed a discordant pattern among the evolution of *SIX* genes and housekeeping genes (*EF-1*α, *RPB1*, and *RPB2*) and also showed strong evidences of horizontal gene transfer (HGT) of *SIX* genes in *Foc*. Findings supporting HGT were recently reported when sequences of *IGS* and *EF-1*α were analyzed. One of the sequences belonging to pathogenic isolates, were identical to the largest group of local nonpathogenic individuals, while all pathogenic isolates had identical sequences of *SIX1* genes (Deltour et al., 2018).

Once penetration occurrs and *Foc* overcomes the first host barriers, the pathogen produces thickened hyphae and microconidia. The thickened hyphae then develop into chlamydospores in intra- and intercellular spaces (Li et al., 2011). A few hyphae can be seen in xylem vessels and growing in the root cortex before 10 days after inoculation (Li et al., 2011, 2017; Xiao et al., 2013). A hyphae network develops in the intercellular spaces along the junctions of root epidermal cells and also could be observed in xylem of the rhizome after some days (Li et al., 2011; Guo et al., 2015). Once *Foc* reaches the vascular zone of the lateral roots, the rhizome infection will occur (Figure 2C). *Foc* colonization of the rhizome vascular bundles occludes the vessels interfering with nutrient uptake and upward water transport to the pseudostem and leaves (Li et al., 2017). Rhizome infection is the most important step to disease development (Li et al., 2017). Once the rhizome was colonized the infection becomes systemic reaching the pseudostem (Figures 2D,E). According to Xiao et al. (2013), a large amount of hyphae is observed in the pseudostem at 17 days after the inoculation (dpi) and the plant may die at 24 dpi.

The infection process investigated under controlled conditions seems to reflect what happens in the field, but disease development and symptom expression may vary depending on different factors (see **Factors driving disease intensity**) such as cultivar resistance. Under natural conditions, the disease is mostly perceived at flowering (Figure 2D). However, highly susceptible cultivars in the presence of high inoculum pressure may show external symptoms as early as 3 months after planting. Quick disease development has been observed in *Foc* R1 in Silk (AAB) in Brazil, *Foc* R2 in Bluggoe (ABB) in Nicaragua and *Foc* TR4 in Cavendish (AAA) in Taiwan. Apparently, under natural conditions, most conidia and chlamydospores produced by *Foc* return to the soil when the plant dies (Figure 2F). Chlamydospores may remain dormant or in secondary hosts for several years (Figure 2A) or start a new disease cycle (Figure 2A) immediately when favorable conditions and a susceptible host are available (Stover R., 1962). The perennial monocrop system used for banana is propitious for continuous FW cycles and an increasing inoculum build up.

## Pathogen dispersal

Apparently, *Foc* does not spread in soil by active vegetative growth as other soil-borne pathogens, such as *F. solani, F. pallidoroseum, Rhizoctonia* sp. and *Pythium* sp. (Stover and Waite, 1960; Stover R., 1962; Trujillo and Snyder, 1963). Dispersal mainly takes place by passive movement of pathogen propagules at short and long distances, from farm to farm or other locations locally or between countries or continents. Long distance dispersal is mainly due to anthropogenic-related factors, while dispersal at short distances may be associated to both anthropogenic and natural factors, such as water runoff, animal movement or spore-bearing soil.

**Figure 3:**
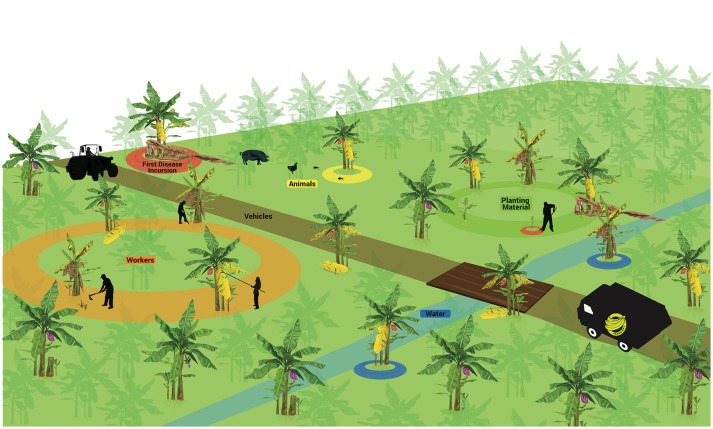
Factors associated to pathogen spreading in Fusarium wilt epidemics in bananas. First incursion (upper left). Vehicles (middle). Planting material (upper right). Animals (upper left). Workers (bottom left). Water (in blue). These factors may operate separately or in association to disperse *Fusarium oxysporum* f. sp. *cubense* structures short or long distances.

The few studies on the spatial dynamics suggest that randomly distributed infected plants can be found in the field at the onset of the epidemics, but thereafter the main process for disease dissemination in the field is plant-to-plant movement, which ultimately leads to the aggregated pattern commonly seen in many areas (Meldrum et al., 2013). As a soil-borne pathogen, agents capable of moving soil particles and spores in the soil, including water, contribute significantly to pathogen spread. The movement of plant parts, including planting material, also appears to have a prominent role. A summary of main dispersal agents is detailed in Figure 3 and discussed below.

### Plant materials

Once a plant is infected and colonized by *Foc*, production of chlamydospores in the root system, rhizome, and pseudostem is triggered (Li et al., 2011). Pathogen structures have also been found in the petiole and the pathogen DNA was also detected in leaves (Lin et al., 2009). Once a *Foc*- infected plant completes its cycle or is killed by *Foc*, these structures also die. While the aboveground parts and the rhizome can be readily destroyed, the roots remain in the soil. Therefore, if *Foc* was actively growing and colonizing the root system, spores could remain in the soil and become part of the arsenal of pathogen inoculum. Studies testing the role of banana roots on short-distance dispersal of *Foc* are needed to elucidate the possible involvement of banana roots as inoculum reservoir. These findings may have direct impact on crop and disease management.

Infected mother plants are able to transfer fungal structures to the suckers, which typically remain symptomless due to the long latent period of the disease (Stover R., 1962). Therefore any infected plant material, including symptomless suckers, is a potential inoculum source and can also disperse the pathogen. Planting material has been (and continue to be) one of the most important factors for FW dissemination (Ploetz, 2005; Dita et al., 2010; Pérez-Vicente et al., 2014). Additionally, banana leaves and pseudostem are frequently used for wrapping or packing banana. Infected plants used in compost preparation result in contaminated substrate which is readily transported from place to place.

As yet, no scientific evidence has been published of dispersal of *Foc* in banana fruits, but better understanding of this risk is required. A recent study related to the presence of *Foc* TR4 in the Middle East included the analysis of this possibility (Ploetz et al., 2015). A publication from Australia indicates that *Foc* could move as both symptomless infection of the vessels in fruit crowns, and in pieces of infected leaf trash associated with fruit shipments from The Philippines (Commonwealth of Australia, 2004). In summary, the movement of plant parts either to be used as propagation material or as a result of agricultural practices can imply significant risks at local, regional, and continental levels and should be strictly controlled in *Foc*-affected areas.

### Water

Heavy rainfall hits the soil and drops containing contaminated fragments of infected plant materials and soil particles can splash causing short-distance *Foc* dispersal. Likewise, runoff flow may carry soil and plant parts and contaminate new clean areas, drainage canals, irrigation reservoirs and rivers. Once water coming from contaminated sources is used in irrigation or naturally reaches *Foc*-free areas, the pathogen spreads rapidly, and efficiently into and between banana fields. For instance, the rapid spread of *Foc* TR4 in China has been frequently associated to both infected planting material and irrigation water from the Pearl River (Xu et al., 2003). In areas naturally predisposed to flooding, the risk is even higher. Natural phenomena such as hurricanes and typhoons can boost *Foc* dispersion not only by direct spreading of *Foc* to the banana plantations, but also by contaminating irrigation water sources as recently observed in the Philippines (Trueggelmann, Unifrutti/Bloom Agro, *personal communication*). In this sense, an indirect effect of water might also occur when tissue-culture (TC) plants under acclimatization in greenhouses are irrigated with *Foc*-contaminated water. These TC-plants, though symptomless, might be infected and effectively spread the pathogen to the field. Therefore, TC-plants providers should enforce a strict quality control of water sources. This should be not only applicable for water, but also for TC-plant substrates as they could also be *Foc*-contaminated. Therefore, the whole acclimatization process, including water and substrate quality, should be reviewed to certify TC-planting material is *Foc*-free.

### Soil and substrates

Dispersal of *Foc* in soil is associated with vectors of soil movement. Roots or other debris of infected plants, water, wind, insects, animals, tools, machinery tires, and other equipment can carry soil particles containing *Foc* propagules. Studies in other pathosystems indicate that *F. oxysporum* does not move significant distances in soil without the presence of a host roots, plant materials or other dispersal agents (Trujillo and Snyder, 1963; Rekah et al., 1999). Recent research has estimated the amount of *Foc* TR4 spores in soil samples from the Philippines and demonstrated that it is possible to detect the presence of significant inoculum levels in soil adhered to shoes after a short field visit (Gert Kema, *personal communication*). Substrates, organic amendments or even plants other than bananas may disperse *Foc*. For instance, in the coffee zone of Central America where bananas are associated with agroforestry coffee, *Foc* R1 is dispersed with coffee seedlings prepared with *Foc*-infected substrates. The growing trade of substrates, including those with coconut fibers produced in Asia, is potentially risky for the dispersal of *Foc* at transcontinental levels. Further studies of population dynamics of *Foc* in the soil at different layers are necessary to better understand how the pathogen survives, spreads, and infects plants in substrates.

### Wind

The hypothesis that winds accompanied by rains can disperse *Foc* has not yet been confirmed. In dry regions, wind carrying contaminated dust particles could also be a dispersal agent of *Foc*. While aboveground sporulation has not yet been reported in banana, it is necessary to consider this epidemiological factor. Field-based studies on the aerobiology of FW are urgently needed to verify not only the formation of external spores, but also its mode of infection. The use of spore traps was useful to capture airborne conidia of *F. oxysporum* f. sp. *radicis-lycopersici* (*Forl*) and understand its epidemiology in tomato (Rekah et al., 2000). A similar approach could be used to assess the airborne spread of *Foc* and its role on FW epidemiology. In fact, aerial conidia production has been already reported in other soil-borne *formae speciales* of *F. oxysporum*, such as *F. oxysporum* f. sp. *lycopersici* (tomato; *Fol*), *F. oxysporum* f. sp. *cucumerinum* (cucumber) and *F. oxysporum* f. sp. *basilici* (basil; *Fob*). The capacity of airborne spores to cause above ground infections is either unclear or controversial. Experimental inoculation of stem wounds with conidia of *F. oxysporum* f. sp. *cucumerinum* failed to establish infections on cucumber, suggesting that airborne spores are deposited on the substrate surface and infection occurs primarily through the root (Scarlett et al., 2015). In contrast, infection through leaf wounds by airborne propagules of *Forl* in tomatoes and *Fob* in basil have been reported (Rekah et al., 2000).

While the production and dispersal or aerial inoculum of *Foc* under field conditions, as well as its potential to infect banana through above ground tissues need to be further investigated, heavy winds, either associated or not with rain, can effectively transport contaminated soil particles and plant debris from infested to disease-free areas. Therefore, wind should be considered as a dispersal agent when analyzing FW epidemics.

### Animals

A range of vertebrate and invertebrate animals may inhabit banana plantations. These animals can transport soil particles from infested to *Foc*-free areas. Banana weevil borer, *Cosmopolites sordidus* (Germar; Coleoptera: Curculionidae), widespread in banana plantations, is the most important insect pest of bananas and plantains (Gold et al., 2001) and recent studies in Australia demonstrated the presence of viable spores on their exoskeletons (Meldrum et al., 2013). Although this finding means that *C. sordidus* may be a carrier of *Foc*, further studies are needed to determine whether weevils are effective vectors of *Foc. Metamasius hemipterus* L. (Coleoptera: Curculionidae), a false weevil frequently found in banana fields might also have a role on *Foc* dispersion, but no study was found on this subject. In smallholder farming systems or plantations close to human settlements, domestic animals could also contribute to farm-to-farm dissemination of FW. For example, since the detection of *Foc* TR4, rigorous campaigns to reduce feral pig populations have been implemented in the Tully Valley, Australia, as these animals are recognized as a serious vector of soil-borne fungal diseases (Biosecurity of Queensland, 2016). The potential role of subterranean vertebrate pests as *Foc* disseminator has not been studied, for example, pocket gopher, one of the most important vertebrates in banana fields in Central America (Monge-Meza, 2011). In addition, the putative role of other insect vectors needs further attention. For instance, fungus gnats (*Bradysia* spp.) are vectors of *Fusarium* spp. in greenhouses and nurseries in ornamentals (Gullino et al., 2015) and tomatoes (Gillespie and Menzies, 1993). Likewise the implication of shore flies (*Scatella stagnalis*) in the transmission of *Fol* and *Forl* to tomato has been reported (Matsuda et al., 2009). Finally, as a biosecurity rule, mainly for quarantine-declared areas, fencing preventing the transit of animals and the other means of dissemination need to be implemented to reduce risks.

### Anthropogenic factors

Humans play a key role dispersing plant pathogens and *Foc* is not an exception. The current globalized world increases movement of goods, people and trades. Therefore, the risk of introducing *Foc* through anthropogenic factors is clearly high. Rapid dispersal of *Foc* by asymptomatic, but infected, suckers as planting material was crucial for the spread of *Foc* R1 that devastated Gros Michel bananas in the past century (Stover R. H., 1962; Ploetz and Pegg, 2000; Pérez-Vicente, 2004). Likely, suckers are the main factor involved in *Foc* dispersal between nearby farms. Recent transcontinental jumps of *Foc* TR4 from Southeast Asia to Africa or Middle East suggest that anthropogenic factors may be involved. Either the introduction of infected planting material or boots or tools contaminated with *Foc*-infested soil may have been responsible for this process (Ploetz et al., 2015). All farm equipment, clothes, footwear, tools, containers, etc., which had been used in *Foc*-infested areas could transport and spread the pathogen into disease-free areas. The aforementioned is also applicable at farm scale. Unfortunately, in most cases growers do not prevent disease dissemination on a daily basis, either by lack of knowledge or by the lack of capacity or resources. Once *Foc* reaches a disease-free farm, epidemics may quickly develop. Similar dynamics of spread, seen with *Foc* R1 in the last century, are being repeated today for *Foc* TR4 (Ordoñez et al., 2015; Ploetz et al., 2015).

## Factors driving disease intensity

Diverse biotic and abiotic factors may accelerate or slow down *Foc* infections and consequently FW epidemics. Obviously, host resistance and pathogen aggressiveness are two key factors driving epidemics. Unfortunately, comparative studies considering the diversity of both *Foc* and banana germsplasm, in the same environmental conditions and production systems are scarce.

## Biotic factors

### Nematodes

As a soil-borne pathogen that can penetrate the host by wounds, any external factor promoting root damage may facilitate *Foc* infections. In this sense, attack of plant parasitic nematodes may boost FW epidemics in banana. However, in spite of the importance of the burrowing nematode (*Radopholus similis*) as a banana constraint, studies to assess the interaction between *R. similis* and *Foc* at field level are limited, probably due to the fact that *R. similis* became a serious problem only after the introduction of Cavendish cultivars, which are resistant to *Foc* R1. Under greenhouse conditions (Somu, 2012; Dinesh et al., 2014) the combined inoculation of *R. similis* and *Foc* increased the incidence and severity of FW. However, this positive interaction between these pathogens was not observed in another greenhouse study (Chaves et al., 2014). The intensity of FW in Gros Michel bananas was not influenced when co-inoculated with *R. similis* and *Foc* R1. However, co-inoculated plants showed a significant root weight reduction compared with plants only inoculated with *Foc* or *R. similis*. The high susceptibility of Gros Michel to *Foc* R1 may have prevented detection of additional effects. Therefore, the analysis of the role of nematodes in FW epidemics should also consider cultivar resistance to *Foc*. Until more data are available on *Foc*–*R. similis* interaction at field level, the impact of nematodes should not be ignored as they can cause severe damage in banana. Production areas where both *Foc* TR4 and *R. similis* are present offer good opportunities to better understand the role of this nematode on FW epidemics in banana at field level. Possible interactions of *Foc* with other genera of nematodes should also be examined. In some banana production areas in Brazil, *Meloidogyne, Helicotylenchus, Rotylenchus*, and *Pratylenchus* are more prevalent and destructive than *R. similis* (Almeida et al., 2018). Larger populations of *Pratylenchus* spp., migratory endoparasites, were found in areas highly affected by FW. Almeida et al. (2018) suggest that nematode wounds to the roots may facilitate infection and colonization of *Foc*. Studies in other crops show that nematodes can boost FW epidemics. Races 1 and 2 of *F. oxysporum* f. sp. *vasinfectum* (*Fov*) are particularly devastating in cotton when the root-knot nematode, *M. incognita*, is present (Garber et al., 1979). In the absence of nematodes, these *Fov* races cause mild disease (Jorgenson et al., 1978). Likewise, root-knot nematodes increase severity of FW *F. oxysporum* f. sp. *niveum* (*Fon*) in watermelon (Martyn, 2014) and the tobacco cyst nematode (*Globodera tabacum*), has also been associated with higher severity of FW (*F. oxysporum* f. sp. *nicotianae*) in tobacco (LaMondia, 2015).

### Weevils

*C. sordidus* and *M. hemipterus*, may also act as a predisposing factor for FW epidemics, but, as with the nematode-*Foc* interaction, this arthropod-*Foc* interaction also needs further analysis beyond the general assumption that weak plants are more susceptible to *Foc*. Comparative analysis between FW-infested and FW-free areas regarding population density and rhizome damages could help to better understand the influence of weevils attack on FW in banana. In addition, the putative role of weevils on *Foc* dissemination may also impact epidemic as previously discussed.

### Soil and plant microbiome

Since soil suppressiveness was conceived and documented, soils with an active and functionally diverse microbiota are assumed to have a higher capacity to suppress FW (Doran et al., 1996). In contrast, soils with poor biological activity and unbalanced food web would be more conducive. A suppressive soil is that in which the pathogen does not cause high levels of disease or no disease occurred, even in the presence of the pathogen, a susceptible host and appropriate environmental conditions. On the other hand, in a conducive soil even low levels of pathogen inoculum can cause serious damages.

In the last five years, with the advent of more powerful tools to evaluate soil microbiome and its functional diversity, significant progress on banana-*Foc* interaction with the microbiome has been made. Shen et al. (2015) studying suppressive soils to FW of banana in the Hainan Island, China, found higher richness and diversity indices as well as more operational taxonomic units in the suppressive than in the conducive soils. *Chthonomonas* spp., *Pseudomonas* spp. and *Tumebacillus* genera were significantly enriched in the suppressive soil. Likewise, a study also performed in Hainan by Xue et al. (2015), identified *Bacillus* spp. as the most dominant bacterial group isolated in a *Foc*-suppressive soil followed by *Rhizobium, Bhargavaea, Pseudolabrys*, and *Sinorhizobium*. Recently, Köberl et al. (2017) studying healthy versus *Foc*-infected banana plants in Central America, found higher richness and diversity of *Gammaproteobacteria* in healthy plants. In addition, healthy plants also revealed an increase in potentially plant-beneficial *Pseudomonas* and *Stenotrophomonas* species.

As previously stated *Foc* may be dispersed through infected planting material. Therefore, the use of TC-planlets is recommended to reduce this risk. However, TC-plantlets have been shown to be more susceptible to FW than conventional suckers (Smith et al., 1998). One hypothesis to explain this behavior is that during the TC-process beneficial microorganisms are removed, leaving plantlets more vulnerable. Lian et al. (2008) demonstrated that *Bacillus* spp. and *Pseudomonas* spp. were induced upon *Foc* inoculation and were able to colonize and increase the protection of bananas roots against this pathogen. Thus, the soil and plant microbiome may affect FW epidemics either by creating *Foc*-suppressive environments in the soil or by hindering host penetration and colonization. The identification of key microorganisms is proposed as a first step to rebuild the microbiome of TC-banana plants prior to planting, not only to improve defense responses against *Foc* (Forsyth et al., 2006; Weber et al., 2007), but also against nematodes (Vu et al., 2006) and to promote plant growth (Ting et al., 2012).

As aforementioned, there are several factors that might reduce or promote FW in banana including the resistance level of the cultivars, which was not discussed in this section. *Foc*-banana is a complex and multifactorial interaction. Thus, factors like pH, P-content or N-sources may reduce FW intensity in some cases, but only slightly affect disease intensity in other situations such as when affected by a virulent *Foc* variant, in the presence of nematodes in high numbers or even in an environment that favors *Foc* dispersion. There is an urgent need for research in these different aspects of the interactions related to FW epidemics in banana.

Finally, the impact of the environment should not be ignored. For instance, why do some *Foc* populations (*Foc* SR4) only affect Cavendish clones in the subtropics? A common assumption is that cold reduces banana plant defenses (Moore et al., 1993; Ploetz et al., 2015), but which defense genes are affected? What is the role of other factors, such as the decomposition rate of organic matter? Are antagonist soil microorganisms less competitive seasonally in the subtropics? Is the microbiome functional diversity compromised over the cold season in the subtropics? Answering these questions might help a better understanding of FW epidemics of banana both in tropical and subtropical conditions.

## Abiotic factors

Physical and chemical soil characteristics can also influence disease intensity, making the soil suppressive.

### Physical properties

The physical structure of soils has been associated with FW in banana, but so far comparative studies are scarce. In general, well-drained and aerated soils are assumed to reduce FW by improving root development and microbial activity (Stover and Simmonds, 1987). In contrast, soils with high levels of compaction and lower aeration may favor FW. However, physical indicators distinguishing conducive and suppressive soils to *Foc* need to be better understood. Studies conducted by Domínguez et al. (2001) in the Canary Islands indicated that higher values of water-stable aggregates were associated with conducive soils, whereas high clay content was consistently higher in suppressive soils. On an agroforestry farm in Brazil, higher clay content was correlated with higher suppressiveness of soil patches to *Foc*, while sand and silt with conduciveness (Deltour et al., 2017). In contrast, in India, sandy loam or sandy clay loam types of soils with low bulk density are more suppressive to FW of banana, while clay soils with high bulk density are more conducive (Felcy-Navajothy et al., 2012). Recent studies comparing clean and infested areas reported an association between higher values of soil penetration resistance and soil density with higher FW intensity in banana. Although studies in different sites have generated contrasting results as seen above, the role of soil physical properties should continue to receive attention by both researchers and growers.

### Chemical properties

Synthetic fertilizers have impacted global agriculture since the green revolution and the banana crop is not an exception. The source and levels of these compounds can not only influence yield, but also the intensity of diseases (Huber and Watson, 1974) and in the case of soil-borne pathogens like *Foc* this interference may be more complex because of the direct impact on the pathogen habitat. Soil pH, which is influenced by many factors, is a fundamental variable. Higher levels of FW in banana are consistently associated with lower pH values (Domínguez et al., 2001; Nasir et al., 2003; Deltour et al., 2017). In fact, practices that reduce soil pH values, such as the application of urea and ammonium as sources of nitrogen (N) have been historically associated with severe epidemics of FW in bananas (Sequeira, 1958; Stover R., 1962; Nasir et al., 2003). However, questions on whether lower pH values cause a shift in the soil microbiome, interfering with plant resistance or enhancing pathogen virulence have been raised. In this scenario the type of N source, ammonium or nitrate, plays a fundamental role. Nitrate (${\text{NO}}_{3}^{-}$) generally increases the pH near the rhizosphere, whereas ammonium (${\text{NH}}_{4}^{+}$) reduces it. It is also generally assumed that ammonium applications boosts FW epidemic, whereas nitrate reduces it (Mur et al., 2016). However, the differential effects of these N sources on FW are not solely due to the impact on soil pH. According to Dong et al. (2016), nitrate contributes to increase the lignin content in banana after *Foc* infection and also improves the absorption of resistance-related nutrients thereby maintaining a higher photosynthetic rate and high disease resistance. In contrast, ammonium keeps the lignin content relatively stable and does not improve nutrient uptake. In addition, the effect of urea and other ammonium-based sources of N on increasing FW might also be related to citrate regulation (Wang et al., 2016). These authors associated the regulation of citrate exudation with the increase of FW intensity in cucumber. Nitrate significantly suppressed the disease compared with ammonium. Interestingly, in the ammonium-treated plants, citrate enhanced pathogen spore germination and penetration, increasing both disease incidence and pathogen population (Wang et al., 2016). While the impact of these N sources in the microbiome remains to be better understood in banana, the hypotheses that ammonium also suppresses population density of bacteria in soils deserves more attention. In summary, the available data so far indicate that lower pH values and ammonium-based sources of N increase FW in banana. However, changing pH on soils suppressive to FW in banana had little effect on disease severity (Peng et al., 1999). Therefore, the relation of N (sources and levels) and pH need to be addressed in management strategies (see **Management practices oriented to soil health and suppressiveness**).

Phosphorous (P) has been shown to be very important on root development and consequently may have direct implications on soil-borne pathogens like *Foc*. Comparative analyses of infested and clean areas in Brazil revealed that low soil P availability was associated with high FW incidence (Furtado et al., 2009). Interestingly, higher P levels were also correlated with FW suppression in banana fields in China (Shen et al., 2015). As P plays an important role in root development in banana, ensuring adequate P over the crop cycles (mainly at planting and in management of ratoon suckers) seems essential for better FW management.

Banana is a highly potassium-demanding crop, but consistent data on the role of potassium (K) on FW intensity, other than K-deficient plants are more susceptible to diseases, is lacking. In other crops, such as cotton, oil palm, tomato and muskmelon, relevance of K in reducing FW has been documented (Perrenoud, 1977). Long-term experiments to decipher the role of K on FW in bananas need to be conducted, as high K levels are always recommended at flowering, when, coincidentally, FW symptoms are more evident.

Applications of calcium (Ca) and magnesium (Mg) seem to reduce FW in banana and the effects are commonly associated to increasing pH values. However, adding CaCO_3_, Ca(OH)_2_ or CaSO_4_ to the soil reduced the germination of chlamydospores and FW severity in banana without changing soil pH (Peng et al., 1999). In addition, Furtado et al. (2009) also found that Ca and Mg levels in the soil were significantly lower in banana areas affected by FW when compared with healthy sites, without any relation with pH.

Silicon (Si) application can also reduce FW symptoms in greenhouse conditions (Fortunato et al., 2012a). The reduced intensity of FW in Si-treated plants was correlated with higher concentrations of hydrogen peroxide (H_2_O_2_), total soluble phenolics and lignin-thioglycolic acid derivatives and greater activities of enzymes, like as phenylalanine ammonialyases, polyphenoloxidases, peroxidases, β-1,3-glucanases, and chitinases (Fortunato et al., 2012b). While a clearer effect of Si on the reduction of FW at field levels remains to be demonstrated, the application of Si should be considered for bananas at pre-planting as it can improve both the nutrient balance and boost plant defenses against pathogens (Wang et al., 2017).

The role of micronutrients in promoting or reducing FW in banana is less clear and in some case contradictory. According to Domínguez et al. (2001), available iron (Fe) is more abundant in soils where FW of banana is more severe in Canary Islands. The same authors suggested that higher content of Fe might promote *Foc* spore germination and increase disease severity. However, adding Fe-EDDHA (Fe 6%) to the soil reduced germination of *Foc* and FW severity in banana experiments conducted in Australia (Peng et al., 1999). The potential role of siderophore-producing bacteria, such as *Pseudomonas* spp., to reduce chlamydospores germination of *F. oxysporum* f. sp. *cucumerinum* has also been reported (Simeoni et al., 1987).

Fusarium wilt appeared to be more severe in bananas growing under Zn deficient conditions (Borges-Pérez et al., 1983, 1991). A possible role of Zn on improving tylose formation was suggested (Borges-Pérez et al., 1983). However, experiments conducted with *Foc* SR4 in Canary Islands failed to show a response (Hecht-Buchholz et al., 1998). These authors suggest that alterations in the ultrastructure of chloroplasts and mitochondria may be the link between Zn deficiency and FW intensity.

In general macro- and micro-nutrient deficiency or inadequate use could be linked to high FW intensity, but also to many other diseases (Mur et al., 2016). Isolating single nutrient responses or interactions in fields with patchy *Foc* presence and variable soil biotic conditions remains a challenge.

## Options to manage fusarium wilt of bananas

Considering the epidemiological aspects of *Foc* and the perennial and monoculture nature of most banana plantations, it is evident that FW management is not simple, unless a resistant and commercially accepted cultivar is available. The use of resistant cultivars is frequently stated as the only effective measure to manage this disease (Ploetz, 2015a,b). However, resistant cultivars might not match market's demands and resistance may be overcome by new pathogen strains, as is the case of Cavendish and *Foc* TR4. Unfortunately, the “dogma” on the effectiveness of cultivar resistance as the only plausible option to manage FW in banana may lead to insufficient emphasis on exclusion, biosecurity, soil management, as well as, innovative alternative options to reach integrated and long-term disease management approaches. Some of these measures can avoid or delay disease epidemics, reduce disease intensity and also enhance yields. Below we discuss a set of field options to manage FW of banana from exclusion to integrated disease management approaches, considering not only aspects of FW epidemiology, but also practices to enhance crop production.

### Exclusion

Pathogen exclusion is a key measure to manage plant diseases, particularly those that do not occur in a given area, such as is currently the case with *Foc* TR4 in Latin America and the Caribbean (LAC), a large part of Africa and even in countries in Asia where TR4 has recently been detected. Therefore, major emphasis must be given to preventive measures at plot, farm, country, regional, and continental levels to avoid the entrance of pathogens. Exclusion has gained increasing interest to prevent or delay the entrance of the highly destructive *Foc* TR4. In this sense in LAC, the potential impact of *Foc* TR4 has been discussed in many technical events and action plans have been proposed to avoid the entry of this strain (Dita et al., 2013). However, exclusion and quarantine measures are extremely dependent on diagnostic tools, awareness, preparedness, readiness and a legal framework supported by National and Regional Plant Protection Organizations (N/RPPOs).

Different diagnostic methods to identify *Foc* TR4 (VCG 01213/16) are currently available and have supported the decision-making process of plant protection officers worldwide. Some of these methods are specific for VCG 01213/16, which is the strain globally recognized as *Foc* TR4 (Dita et al., 2010; Zhang X. et al., 2013). However, other tools detect more than one VCG. For instance, the methods described by Lin et al. (2009, 2016) react with eight different VCGs, in addition to 01213/16 (Dita et al., 2010). The method proposed by Aguayo et al. (2017) detects VCG 01213/16, but also VCG 0121. While VCG 0121 and 01213/16 are genetically related, it is important to consider that VCG 01213/16 is currently the only widely recognized as *Foc* TR4, and more importantly, the only one officially listed as a quarantine pest by many N/RPPOs worldwide. In this sense, regulatory agencies need to verify carefully each diagnostic tool and conceive the diagnostic as a process with different steps that starts in the field (adequate samples, expertise on the disease) and is later complemented with different laboratory techniques as described by O'Neill et al. (2016).

In spite of the importance of *Foc* TR4, FW epidemics in banana are not caused only by this race. There are non-TR4 strains currently causing serious epidemics and yield losses in Nicaragua, Peru and Brazil. Exclusion should also be implemented within country in these situations. Unfortunately, rapid and efficient tools to detect these strains are still missing. A generic diagnostic method to detect any banana-pathogenic strain of *Foc*, independently of the VCG to which it belongs, would improve biosecurity and quarantine measures to support epidemiological studies and consequently management tactics. While these diagnostic tools are not available, biosecurity measures should be implemented from country borders to the farm gates and should go further, not only targeting *Foc* TR4, but other pests and diseases of socio-economic relevance.

Certification agencies will play an important role to reduce the entrance and spread of *Foc* TR4 with a requirement for biosecurity measures at farm level as recently implemented by Global Gap (https://www.globalgap.org/uk_en/for-producers/globalg.a.p.-add-on/tr4-biosecurity/).

### Eradication of infected plants and pathogen containment

Many practices to eradicate *Foc* have been tested, but there are no reports on complete elimination of the pathogen. Therefore, once *Foc* is established in a field, management strategies must be focused on avoiding the spread of the pathogen to disease-free areas and decreasing the level of inoculum in the infested area. Destruction of infected plants to reduce inoculum build-up and prevent pathogen spread is a fundamental starting point. Efficient inoculum reduction of *Foc* has been achieved by treating infected plant materials with urea under anaerobic environments (Biosecurity of Queensland, 2016).

Even as infected plants are being destroyed, the analysis of risk of pathogen movement into new areas should be detailed (see **Pathogen dispersal**). Strict biosecurity practices at the farm's gate and around the complete perimeter are essential to lock down contaminated areas. The following steps can support containment efforts: (1) Minimize the access of outsiders to farms and packing houses with zero access to areas where risk is highest, (2) Shoes and tools used by employees and visitors should remain in the farm, (3) Implement practices to minimize movement of soil and water from contaminated areas, (4) Foot and vehicle baths with proper disinfectants available on strategic points in the banana plantation and packinghouses; (5) Build capacities on disease diagnostic, epidemiology and biosecurity among all persons associated with farm, and (6) Maintain communication channels with biosecurity officers and NPPOs to report any new suspicious plant or unexpected violation of containment.

### Resistant cultivars

Once FW is established in the area, the use of resistant varieties is the most effective means to manage this disease. The resistance of Cavendish to *Foc* R1 has had an enormous impact on the banana industry contributing to near complete Cavendish dominance in export trade. This resistance has been effective for about 50 years and it is an iconic case of resistance durability in crops to pathogens. In general, resistant cultivars are effective for less than 10 years (Johnson, 1984; McDonald and Linde, 2002). To date, there are no commercial cultivars resistant to *Foc* TR4 with similar levels of resistance of Cavendish to *Foc* R1.

To better understand the levels of resistance and its implication on FW management of bananas, three terms, considering the presence of *Foc* and favorable environmental conditions, are discussed below.

#### Complete resistance

This definition, also called, qualitative resistance, is illustrated by the Cavendish (AAA)-*Foc* R1 interaction. *Foc* R1 does not cause any physiological disturbance, does not affect yield and there is no increase in inoculum levels in the field.

#### Intermediate resistance

Also referred to as quantitative resistance, cultivars with intermediate resistance show less severe symptoms or damage than susceptible varieties when grown under similar environmental conditions and inoculum pressure. The Prata (AAB)–*Foc* R1 interaction can illustrate this definition. In this case, the inoculum density might be driving the intensity of FW. Prata can resist the infection by *Foc* R1 only up to certain levels of inoculum density. Under environmental conditions and management practices favorable to *Foc* (see **Factor driving disease intensity**) FW and yield losses will increase gradually.

#### Susceptibility

This definition can be illustrated with different interactions, such as Gros Michel-*Foc* R1, Cavendish-*Foc* TR4 and Silk-*Foc* R1. Severe epidemics of *Foc* TR4 in Cavendish and *Foc* R1 in Silk are often recorded and plantations may be totally destroyed in <2 cropping cycles. In these varieties, *Foc* infects the host and causes serious physiological disturbances and yield losses. Inoculum levels in the soil increase dramatically preventing new plantations of the same cultivar from being re-established in the same area.

Ideally, **complete resistance** should be used. If complete resistance is available, then cultural practices are mainly oriented to increase yield and to control other pests and diseases as has been the case of Cavendish plantations in Asia before the emergence of *Foc* TR4. However, after the emergence of *Foc* TR4, several Cavendish somaclones called Giant Cavendish Tissue Culture Variants (GCTCV) developed in Taiwan (Hwang and Ko, 2004) with **intermediate resistance** to *Foc* TR4 have been planted. Two clones of the first generation of GCTCV, GCTCV-118 and GCTCV-119, and recurrent selection based on the first somaclones, resulted in GCTCV-218 and GCTCV-219 which are being used to mitigate losses caused by *Foc* TR4 in Taiwan and the Philippines and have recently been planted in Mozambique.

Reduction in FW intensity using these somaclones in infested areas where regular Cavendish varieties cannot be grown has been widely communicated in international meetings. Peer-reviewed publications on the status and dynamic of soil inocula and disease incidence in commercial banana fields planted with these somaclones are not available yet. However, as mentioned, resistance to *Foc* TR4 is not **complete**, but **intermediate** (Hwang and Ko, 2004). Therefore, depending on inoculum pressure diseased plants can be observed as early as the first crop cycle. In all cases management practices, such as early detection and destruction of infected plants and a set of biosecurity measures for containment are recommended (see **Integrated management practices**).

Intermediate resistance has an enormous value (Corwin and Kliebenstein, 2017), mainly when no resistant commercial cultivars are available. However, the long crop cycle (> 12 months) and perennial nature of bananas cultivation, bring additional implications for epidemiology and management, especially for growers used to a totally resistant variety, as Cavendish to *Foc* R1. Therefore, having certain levels of FW incidence in these Cavendish somaclones or in any other variety with intermediate resistance requires special attention to: (1) increasing inoculum levels of the pathogen in early infected patches, (2) pathogen dispersion from highly contaminated to less contaminated areas in the same farm and (3) pathogen dispersal from infested to disease-free areas. In fact, survival and inoculum buildup of *F. oxysporum* have been already reported in resistant varieties of other crops. For instance, *Fov*, can survive in the roots of resistant cotton cultivars, contributing to the maintenance of inoculum levels in the field (Cianchetta and Davis, 2015). Resistant tobacco varieties, while not exhibiting wilt disease symptoms, increased or maintained populations of *F. oxysporum* f. sp. *nicotianae* (*Fon*; LaMondia, 2015) and in lettuce, *F. oxysporum* f. sp. *lactucae* (*Fola*) can also colonize resistant lettuce cultivars (Scott et al., 2014). Thus, even when planting a resistant genotype, efforts in exclusion, eradication, containment, and integrated disease management need to be ongoing and more intensive with time.

### Management practices oriented to soil health and suppressiveness

Based on current understanding of FW epidemiology, management practices oriented to soil health and suppressiveness, such as crop rotation, the use of cover crops, application of organic amendments and biocontrol agents, as well as, the use of appropriate inorganic fertilizers and agronomic practices (see **Factors driving disease intensity**) can help suppress *Foc* inoculum, reduce disease intensity and enhance yields. The challenge is to identify the most effective practices for the pathosystem of a soil-borne pathogen with long-term survival capacity affecting a perennial crop with continuous cycles of flowering and harvesting. Promising components in FW management are emerging, although integrated field-scale validation is still incipient (Table 1).

**Table 1:** Soil management practices and their effectiveness to control *Fusarium* wilt of banana under field conditions.

**Management practice**	**Observed effects**	**Country**	**Genotype**	Foc race^*^	**References**
Crop rotation and interplanting (*Manihot esculenta*)	Reduction of disease incidence. Low (less than 5%) incidence was maintained over three cropping cycles.	Indonesia	Cavendish (AAA)	TR4	Buddenhagen, 2009
Crop rotation (*Allium tuberosum*)	Reduction of disease incidence (up to 97 %) and improved crop value (up to 86 %). Antifungal volatiles released by *A. tuberosum* were associated with pathogen suppression.	China	Brazil (Cavendish, AAA)	TR4	Huang et al., 2012; Zhang H. et al., 2013
Crop rotation (*Ananas squamosa*)	Reduction of disease incidence up to 60% when compared with maize. Reduction of *Foc* abundances in the soil. Higher abundances of *Acidobacteria, Planctomycete* and *Chloroflexi* observed positively corresponded to *Foc* reduction.	China	Brazil (Cavendish, AAA)	TR4	Wang et al., 2015
Cover crop (*Arachis pintoi*)	Reduction of disease intensity by 20%. Increased the bunch weight in the second crop cycle.	Australia	Ducasse (Pisang awak, ABB)	R1	Pattison et al., 2014
Organic amendments and bio-organic fertilizers	Different organic amendments (cattle manure compost, pig manure compost Chinese medicine residue compost, bio-organic fertilizer -BIO) were compared with general operation control during one cropping cycle. Plants treated with BIO showed the lower disease incidence (20%) when compared with the control (38%). Pig manure showed highest incidence values. BIO improved soil microbial communities.	China	Brazil (Cavendish, AAA)	TR4	Shen et al., 2013
Bioorganic fertilizers	Continuous application of a bioorganic fertilizer (BIO) reduced the disease incidence (15%) when comparing with the control (40 %) over three cropping cycles. BIO also increases yields (up to 24%) and enriched culturable bacteria (*Firmicutes, Gammaproteobacteria* and *Actinobacteria*), potentially associated with pathogen suppression.	China	Brazil (Cavendish, AAA)	n.d.	Fu et al., 2016
Application of microorganisms	A set of 10 isolates of non-pathogenic *Fusarium oxysporum* (np*Foxy*) to banana reduced significantly the intensity of the disease in the greenhouse bioassays. When these isolates were tested in the field no disease reduction was observed. No disease reduction was observed with use of *Pseudomonas fluorescens* WCS 417 alone or combined with np*Foxy*.	South Africa	Cavendish (AAA)	SR4	Belgrove et al., 2011
Application of botanical formulations and biocontrol agents	Two botanical fungicides (Wanis 20 EC and Damet 50 EC), two, *P. fluorescens* strains (1, Pf1) and *Bacillus subtilis* (TRC 54) were tested individually and in combination under greenhouse and field conditions. Combined application (Wanis 20 EC + Pf1 + TRC 54) reduced disease incidence under greenhouse (64%) and field (75%) conditions.	India	Rasthali (Silk, AAB)	R1	Akila et al., 2011

* *Foc, Fusarium oxysporum f. sp. cubense; n.d., not determined*.

#### Crop rotation

Widely used to manage soil-borne diseases in annual crops, crop rotation may be an option in some situations for a perennial crop like banana. Diversified farms may have access to different crops for crop rotation and intercropping as a strategy to manage FW. In this sense, crops with immediate market opportunities, such as cassava (*Manihot esculenta*), pineapple (*Annanas squamosa*) or plant species with different uses or purposes like Chinese leek (*Allium tuberousum*), have been used with different levels of success (Buddenhagen, 2009; Huang et al., 2012; Zhang H. et al., 2013; Wang et al., 2015; Table 1). However, in other cases crop rotation has not resulted in positive results. The use of velvet bean and sorghum did not contribute to reduce FW intensity as much as that obtained when sugarcane was used as succession crop (Sequeira, 1958). Later, sugarcane was used in combination with fallow with better results (Sequeira, 1962). However, in Taiwan, sugarcane was not recommended as a long-term strategy (Hwang, 1985). Crop rotation might have a direct effect lowering the *Foc* inoculum in the soil by creating a suppressive environment, an indirect effect by reducing or eliminating symptomless weed hosts or both. In most cases, the mechanism involved in the suppression of *Foc* has only been partially elucidated (Table 1; Huang et al., 2012; Zhang H. et al., 2013; Wang et al., 2015). Finally, it is important to consider the ability of *Foc* to colonize other crops. This capacity has been reported for other *forma speciales* of *F. oxysporum*, such as *Fov*, which in addition to cotton, can also infect soybean, flue-cured and burley tobacco, okra, alfalfa and lupine (Cianchetta and Davis, 2015), *Fola*, which colonizes three crops commonly grown in rotation with lettuce: broccoli, cauliflower and spinach (Scott et al., 2014) and *Fon*, that infects both tobacco and sweet potatoes (LaMondia, 2015).

#### Cover crops

The use of cover crops (also called ground cover or cover plant species) is considered a good agricultural practice for banana production and can help to manage weeds, pests, and diseases and also increase yield (Djigal et al., 2012; Pattison et al., 2014). Cover crops can be used before planting as green manure, as inter-planting or even as perennial species cultivated with banana. There are few studies available that assessed the effects of cover crops on FW. Pattison et al. (2014) found that Pinto peanut (*Arachis pintoi*) as ground cover reduced the intensity of FW in Ducasse bananas (Pisang awak, ABB) by 20% (Table 1). In addition, authors described a positive effect on yield through the increment of the bunch weight. In other FW pathosystems, such as in watermelon (Himmelstein et al., 2014) and cucumber (Klein et al., 2011), this practice has been successfully used. However, in other cases no positive effects have been observed (Njoroge et al., 2008). Similarly to crop rotation, cover crops can affect physical, chemical, and microbiological soil parameters and are also influenced by environment. Therefore, the selection of appropriate species or varieties, the planting rate and management need to be determined experimentally in each situation. One should always keep in mind that some cover species can eventually act as a host to *Foc* (see **Pathogen survival**).

#### Organic amendments

Organic matter management is essential for soil health and suppressiveness (Noble, 2011; Larkin, 2015). Although organic matter can be added through crop residues and cover crops, off-field sources, such as organic amendments (OAs) are particularly important as they can be enriched with specific microorganisms (Noble, 2011; Hadar and Papadopoulou, 2012; Zhang et al., 2014). In addition, OAs can be applied at different dosages and in target sites, such as disease hotspots. Yogev et al. (2006) showed that composts based on plant-waste residues suppressed diseases caused by four different *formae speciales* of *F. oxysporum: melonis, basilici, radicis-lycopersici*, and *radicis-cucumerinum*. However, there are significant differences between banana and these annual crops, not only in terms of cropping cycle, but also in the amount of secondary inoculum produced per area. An infected banana plant may produce substantially more secondary inoculum than these annual crops. Therefore, the level of intervention to suppress *Foc* inoculum with application of OAs may need to be greater and integrated with other management practices such as the use of beneficial and antagonist microorganisms. In this sense, the susceptibility of *F. oxysporum* to competition for nutrients in the soil (Hadar and Papadopoulou, 2012) may facilitate its suppression if good competitors are in place. For instance, Fu et al. (2017) reported the suppression of FW in banana by the continuous use of an organic fertilizer. However, the effect of OAs on disease suppression may also be linked to biological control. In fact, the suppressive effect of OAs has increased by adding microorganisms (Table 1). Although the application of OAs is generally assumed to be beneficial to soil health, results can be variable and dependent on many factors (Bonanomi et al., 2010). This practice may also have risks as sometimes quality control of products is lacking (Hadar and Papadopoulou, 2012). For instance, the application of chicken manure increased the incidence of FW of banana in greenhouse experiments (Pittaway et al., 1999; Nasir et al., 2003). This pattern has been also observed in field conditions in Brazil, especially when using chicken manure without proper decomposition. The negative effect of chicken manure has been attributed to increased root damage, lower soil pH and the N source, which acted as predisposing factors (Nasir et al., 2003). In general, the application of composts alone often results in inconsistent levels of disease control (Lang et al., 2012). Therefore, further enrichment of composts with target microorganisms to produce the so-called, bioorganic fertilizers have been tested with promising results (Huang et al., 2011; Lang et al., 2012; Qiu et al., 2012; Zhang et al., 2014).

#### Biological agents

The potential of beneficial microorganisms to control FW in banana has been studied since 1945 (Thaysen and Butlin, 1945; Table 1). Particular efforts have been made exploring the potential of non-pathogenic *F. oxysporum* (np*Foxys*) and *Trichoderma* spp. (Fravel et al., 2003; Forsyth et al., 2006; Belgrove et al., 2011). However, in spite of np*Foxys* being commonly found associated with banana plant (as endophytes) and in the soil, large scale use as a biocontrol agent to manage FW (or other diseases) should be carefully evaluated as some strains can increase FW disease (Forsyth et al., 2006) and horizontal gene transfer may also occur transforming np*Foxys* into pathogens (Ma et al., 2010).

Promising results have been more frequently reported in recent years (Table 1), but no single biological product can be recommended for widespread use to control FW of banana. Many factors could be responsible for the historical lack of success of biological agents to control FW in banana (and other crops). The pursuit of a biopesticide approach where single or strain mixtures are directly applied in the soil certainly underestimates the complexity of soil-pathogen-plant interactions. Managing the soil microbiota has an enormous potential to control soil-borne diseases and suppressive soils may hold key answers to better understand and explore microbes and develop efficient management tools (Weller et al., 2002). New and powerful approaches, such as metagenomics, have provided significant advances in understanding functional plant and soil microbiome and identifying promising microbes (Mendes et al., 2015; Shen et al., 2015; Cha et al., 2016). For instance, studies carried out in affected and unaffected soils showed that suppressive sites harbor unique microorganism communities with higher richness and diversity (Shen et al., 2015; Köberl et al., 2017). While applying “suppressive” organisms as bio-pesticide has largely failed (Mazzola and Freilich, 2017), the combination of target microorganisms with organic amendments has shown better results (Table 1). Shen et al. (2015) found that *Acidobacteria* was more abundant in suppressive soils, while *Bacteroidetes* were more abundant in conducive soils. *Bacillus* was the most abundant genera in the suppressive soil and a *B. amyloliquefaciens* strain (NJN-6) isolated from the suppressive soil showed significant inhibition of *Foc in vitro*. Later, this strain was combined with compost to produce a bioorganic fertilizer, which has shown positive results to reduce epidemic caused by *Foc* TR4 in China (Shen et al., 2013; Xue et al., 2015). In addition, the continuous application of this bioorganic fertilizer changed the composition of the rhizosphere microbial community by increasing bacterial diversity (Fu et al., 2016, 2017). The success of biocontrol agents appears to rely on enhancing microbe persistence and activity (to reduce or avoid successive introductions of target microbes) and recovering the functional diversity of the local (indigenous) microbiota (physical and chemical properties improved). Strategies should rely on a community-oriented approach rather than on a single or a few target microorganisms. Meeting these conditions depend on many factors and require the integration of different tactics as discussed above. Developing disease suppressive and healthy soils takes time and, unfortunately, many farmers operate based on rapid response solutions like the application of pesticides, herbicides or highly soluble fertilizers. However, the benefits of management practices oriented to soil health accumulate across successive years increasing control of pests and diseases and enhancing productivity (Fu et al., 2016).

### Integrated practices

Integrated pest management (IPM) is a sound way to understand and manage the complexity of agro-ecosystems. For a soil-borne pathogen, integrated soil management is an important dimension of IPM in the context of integrated cropping systems management. Nevertheless, identifying the alternative practices to be used and their integration is not an easy task, mainly for those in the forefront of commercial plantations. For instance, control options based on fungicides (Nel et al., 2007) and resistance inducers (Borges et al., 2003, 2004) have been effective *in vitro* and in greenhouse conditions. Field validation is still pending and should address not just short-term disease response, but also impacts on the community of soil and rhizosphere organisms and plant microbiome. This approach needs to be tested broadly in different contexts, taking into account the status of FW (all races), the cultivar diversity and the cropping systems in the geographical area of concern.

**Figure 4:**
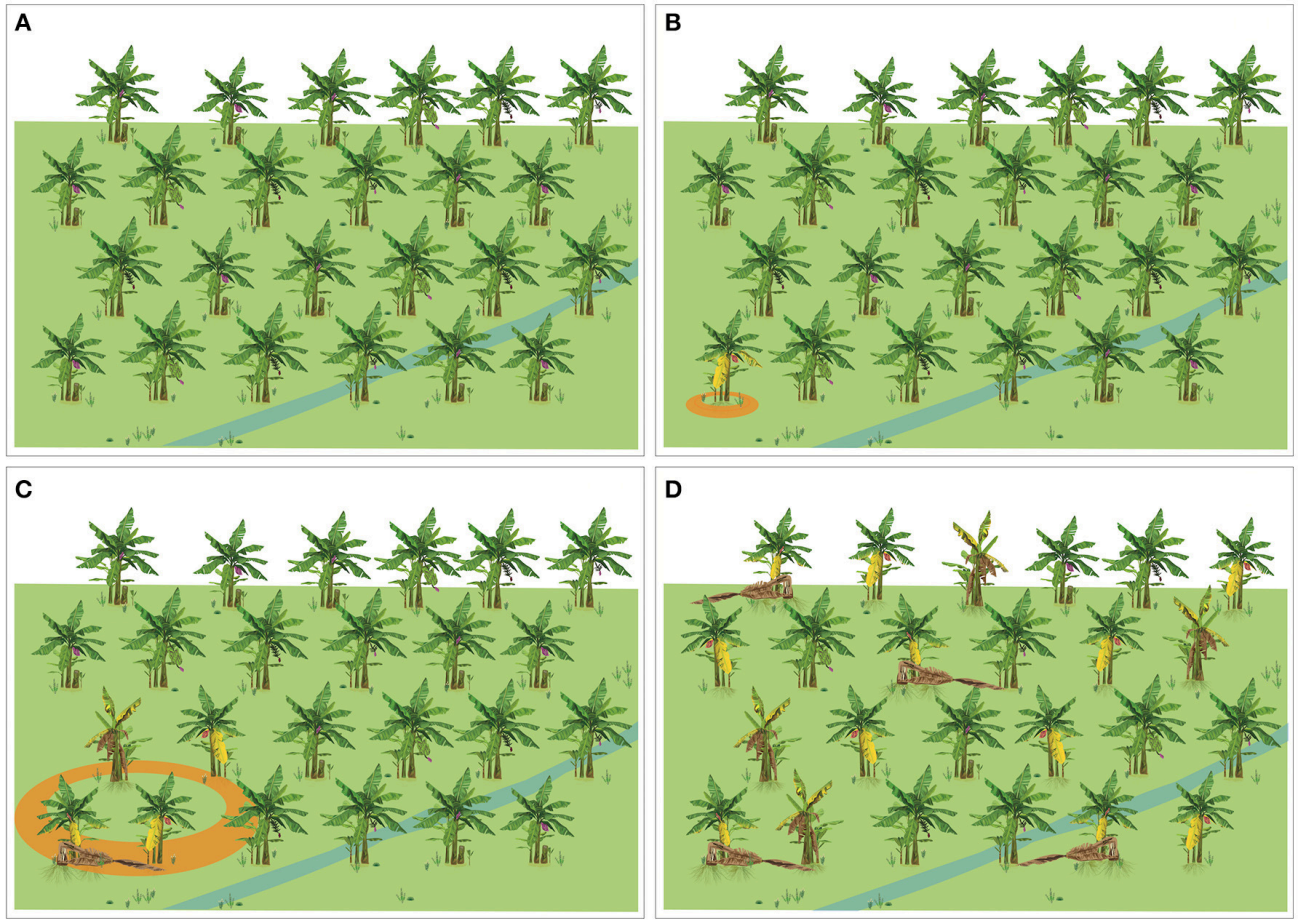
Diagrammatic representation of four different scenarios of Fusarium wilt occurrence in a banana plantation. **(A)** The disease is not present. **(B)** The disease is a quarantine pest and the first incursion was detected. **(C)** The disease is established, but with a patchy distribution and low levels of incidence. **(D)** The disease is evenly distributed at high levels of incidence.

Four different scenarios can be considered at farm level: (a) FW is not present (Figure 4A), (b) *Foc* is a quarantine pest and the first incursion has been detected (Figure 4B), (c) FW is already established, but with low levels of incidence (Figure 4C), and (d) FW intensity is high, the disease is evenly distributed in the plantation and new infected plants are periodically detected (Figure 4D). Considering these different scenarios, management practices and their integration may vary, but some of the practices, such as those aiming at exclusion, pathogen containment and suppression and soil management are applicable to all scenarios.

Exclusion is the preferred management practice, although its success depends on many off-farm factors. Growers need to implement all possible practices to avoid the entrance of any *Foc* strains (or other pests) into their properties. To support these strategies, *ex-ante* and tailored Pest Risk Analyses need to be in place. If eventually an outbreak of FW is detected, then a plan aimed at plant eradication and pathogen containment needs to be activated. Continuous monitoring for early detection is essential. To reach that level capacity building on disease identification and diagnostic is overarching. For instance, how fast can a *Foc* TR4-free country, unequivocally, diagnose, and report a new outbreak of TR4? When detected, are all the players synchronized and resources available to perform plant eradication and pathogen containment? Are national contingency plans available? Are NPPOs connected with independent growers and growers associations to carry out a concerted contingency plan? These questions, among other elemental aspects of biosecurity, need to be clearly answered to guarantee the success of pathogen containment if the first incursion of *Foc* TR4 is detected (Figure 4B).

Once the disease is present (Figure 4C), the use of resistant cultivars (if available) and tactics to suppress the pathogen and boost plant defenses are fundamental. At the same time exclusion and containment should not be neglected as they contribute to further pathogen dispersal and disease intensity, which builds up based on inocula in the soil. Eventually, FW epidemics reach levels where disease management is economically impracticable (Figure 4D). In that situation, plot eradication and crop rotation are inevitable, unless resistant cultivars are available. Finally, management tactics should not only focus on FW, but also sustainable productivity and follow the principle of a continual improvement process.

## General recommendations

### Choice of plots

Fields already infested with FW must be avoided unless a completely resistant cultivar will be planted. The selected area should have adequate drainage. If not, practices to improve drainage need to be implemented. Soil analyses must be performed and nutrients and especially pH must be corrected accordingly.

### Soil pH

Soil with pH values from ranging 5.6 to 6 are recommended. Values for base saturation of Cationic Exchange Capacity (CEC) values must be at least 70%. Special attention must be paid to P, Ca and Mg content.

### Source of N

For applied nitrogen, the use of nitrate sources (i.e., CaNO_3_) is recommended instead of urea or other ammonium (NH_4_) sources.

### Bioorganic fertilizer

Application of organic matter (5 t/ha) supplemented with beneficial microorganisms (i.e., *Trichoderma* spp. and *Bacillus* spp.) is strongly recommended. See options of bio-fertilizers in Table 1.

### Cover crops

The use of cover or inter-planting crops is especially useful to manage weeds and improve soil health (Pattison et al., 2014).

### Planting material

The use of disease-free certified planting material is recommended. Tissue-culture planting material should be well hardened with strong and healthy root systems. Rebuilding the plant microbiome both with endophytes and rhizosphere microorganisms by using beneficial microbes when available has proven to improve the plantlets performance against FW in the field.

### Disease monitoring

Field workers should be well trained on recognizing FW-infected plants at early stages and the risks of spreading the pathogen. Plantations should be monitored periodically to identify suspicious plants. Once FW is confirmed, the eradication of the whole mat is recommended. If *Foc* TR4 is a regulated pest and a suspicious plant is diagnosed as positive, Plant Protection officers should be contacted.

## Summary points and future issues

More support to breeding programs is required to enhance both the identification and utilization of new resistant genotypes. These efforts should bring together new technologies such as gene editing and high throughput phenotyping and take advantage of the current knowledge generated for “conventional” approaches. Generating improved diploids has taken a considerable amount of time and has a remarkable value on understanding FW resistance. The so-called New Generation of molecular breeders might be forgetting fundamental knowledge and putting all efforts on new tools ignoring fundamental cornerstones.

Urgent need for exclusion and biosecurity measures with special attention to the strict use of disease-free planting material and prevention of the movement of contaminated soil and water. This need to be seen from farm gates to transcontinental levels and should not only consider TR4, but also more virulent *Foc* populations across countries. “Farmers and scientists cannot afford to be complacent just because they are growing a so-called resistant variety. Quarantine and clean planting material still need to be strongly adhered to” (Daniells, 2011).

The huge variability of *Foc* and its ability to mutate should not be ignored. Therefore the pathogen populations need to be monitored continuously to identify and manage new and more virulent strains.

The benefits of practices oriented to soil health such as organic amendments, cover crops and biological agents for FW control are incremental and cumulative. They are generally slower acting than other chemicals, but may last longer. These practices might not only reduce FW, but also improve the control of other pest and diseases and enhance productivity (Pattison et al., 2014; Haddad et al., 2018). However it is important to keep in mind that the success of these practices appears to be genotype-dependent and linked to the resistance level to FW.

Strategies for integrated management once the disease is present should consider both boosting plant defenses and suppressing *Foc* propagules in the soil. Practices that reduce the ability of the host to express the genetic potential and respond properly to the pathogen invasion may increase disease severity even if other management practices to suppress the pathogen are in place (Nasir et al., 2003).

## Author contributions

MD designed and conceptualized the structure of the work, wrote and reviewed the manuscript and supervised the design of figures. MB carried out a literature review and wrote initial drafts of some sections. DH carried out a further literature review, reviewed some sections and verified references. EM and CS completed critical reviews of organization, argumentation and writing of the manuscript.

### Conflict of interest statement

The authors declare that the research was conducted in the absence of any commercial or financial relationships that could be construed as a potential conflict of interest.
